# Alternative splicing regulates adaptor protein binding, trafficking, and activity of the Vps10p domain receptor SorCS2 in neuronal development

**DOI:** 10.1016/j.jbc.2023.105102

**Published:** 2023-07-26

**Authors:** Sune Skeldal, Lasse Frank Voss, Jonas Lende, Sarah Broholt Pedersen, Simon Mølgaard, Mathias Kaas, Perline Demange, Andreas Høiberg Bentsen, Marie Fuglsang, Marie Rubin Sander, Henriette Buttenschøn, Camilla Gustafsen, Peder Madsen, Simon Glerup

**Affiliations:** 1 Department of Biomedicine, Aarhus University, Aarhus C, Denmark; 2 Department of Surgical Gastroenterology and Transplantation, Rigshospitalet, University of Copenhagen, Copenhagen, Denmark; 3 NIDO | Centre for Research and Education, Gødstrup Hospital, Herning, Denmark; 4 Department of Clinical Medicine, Aarhus University, Aarhus N, Denmark

**Keywords:** SorCS2, BDNF, alternative splicing, cellular stress, dynein, kinesin, receptor trafficking

## Abstract

The Vps10p domain receptor SorCS2 is crucial for the development and function of the nervous system and essential for brain-derived neurotrophic factor (BDNF)-induced changes in neuronal morphology and plasticity. SorCS2 regulates the subcellular trafficking of the BDNF signaling receptor TrkB as well as selected neurotransmitter receptors in a manner that is dependent on the SorCS2 intracellular domain (ICD). However, the cellular machinery and adaptor protein (AP) interactions that regulate receptor trafficking *via* the SorCS2 ICD are unknown. We here identify four splice variants of human SorCS2 differing in the insertion of an acidic cluster motif and/or a serine residue within the ICD. We show that each variant undergoes posttranslational proteolytic processing into a one- or two-chain receptor, giving rise to eight protein isoforms, the expression of which differs between neuronal and nonneuronal tissues and is affected by cellular stressors. We found that the only variants without the serine were able to rescue BDNF-induced branching of SorCS2 knockout hippocampal neurons, while variants without the acidic cluster showed increased interactions with clathrin-associated APs AP-1, AP-2, and AP-3. Using yeast two-hybrid screens, we further discovered that all variants bound dynein light chain Tctex-type 3; however, only variants with an acidic cluster motif bound kinesin light chain 1. Accordingly, splice variants showed markedly different trafficking properties and localized to different subcellular compartments. Taken together, our findings demonstrate the existence of eight functional SorCS2 isoforms with differential capacity for interactions with cytosolic ligands dynein light chain Tctex-type 3 and kinesin light chain 1, which potentially allows cell-type specific SorCS2 trafficking and BDNF signaling.

In eukaryotes, alternative splicing of multi exon gene transcripts increases and diversifies the proteome from a limited number of genes (1). It allows synthesis of protein isoforms with unique domains and motifs to carry out distinct biological functions (2). This ensures proper expression of critical protein variants most often in a developmental and tissue-specific pattern but also enables the cell to effectively respond to various challenging conditions (3). A commonly used pattern of alternative splicing involves usage of an alternative splice site at the 5′ or 3′end of introns. This enables insertion of additional nucleotides at positions where exons are joined in the mature mRNA rendering translated proteins with functional inserts.

Human *SORCS2* is the product of one such multi exon gene containing 27 exons separated by 26 introns. The SorCS2 protein belongs to the Vps10p domain receptor family of sorting receptors also containing sortilin, SorLA, SorCS1, and SorCS3 (4, 5, 6). In mice and humans, SorCS2 is expressed in distinct regions of the developing and adult central nervous system (CNS) and transiently during the development of multiple organs (4, 7, 8).

SorCS2 consists of a large extracellular domain encompassing the Vps10p domain, a polycystic kidney disease module, a leucine-rich repeat motif, followed by a transmembrane region, and the 60 amino acid intracellular domain (ICD). SorCS2 is synthesized with an N-terminal propeptide, which is proteolytically cleaved by a proprotein convertase, generating single-chain SorCS2. Removal of the propeptide further allows tissue-specific processing into two-chain SorCS2 held together by noncovalent interactions (9). Interestingly, within the CNS, single-chain SorCS2 promotes growth cone collapse of developing axons, whereas two-chain SorCS2 induces apoptosis in Schwann cells within the peripheral nervous system (9, 10). Both activities are exerted in conjunction with the neurotrophin receptor p75^NTR^ and in the presence of proneurotrophins including the proform of BDNF (proBDNF). In the hippocampus, proBDNF, p75^NTR^, and SorCS2 operate together in the induction of long-term depression (11). SorCS2 influences the activity of mature BDNF as well and is critical for BDNF-dependent long-term potentiation by facilitating translocation of TrkB to postsynaptic densities (11). SorCS2 further translocates the N-methyl-D-aspartate receptor 2A subunit (GluN2A) to the cell surface of medium spiny neurons, thereby contributing to the regulation of striatal-mediated motor function (12). Hence, SorCS2 plays important roles in formation, maintenance, and function of neuronal circuits (9, 10, 11, 12), and findings which are further substantiated by genetic associations of SorCS2 with a number of neurological disorders such as Alzheimer’s disease, bipolar disorder, schizophrenia, attention-deficit/hyperactivity disorder, and alcohol withdrawal symptoms (13, 14, 15, 16, 17, 18). In line with this, SorCS2 knockout (KO) mice display several behavioral abnormalities, including hyperactivity, altered response to alcohol, altered anxiety levels, cognitive impairment, and a paradoxical response to amphetamine (9, 11, 19). We here describe the identification of four human *SORCS2* mRNA splice variants differing in a 14 amino acid acidic cluster motif inserted between amino acids 1139 and 1140 and/or a serine residue at position 1104 within SorCS2 ICD. Each of the four splice variants can be posttranslationally processed from a single-chain into a two-chain form, giving rise to a total number of eight SorCS2 protein isoforms. SorCS2 isoform expression varies among tissues and is influenced by physical stressors. Only splice variants without a serine at position 1104 promote BDNF-induced neuronal branching in SorCS2 KO hippocampal neurons. The variants display differential interaction with sorting and motor adaptor proteins (APs), and accordingly possess markedly different trafficking properties. Hence, SorCS2 variants without the acidic cluster motif interact more robustly with clathrin-associated APs AP-1, AP-2, and AP-3. The individual splice variants further show differential interactions with motor APs dynein light chain Tctex-type 3 (DYNLT3) and kinesin light chain 1 (KLC1), the latter only binding SorCS2 isoforms with an acidic cluster insertion. Hence, SorCS2 alternative splicing regulates SorCS2 subcellular trafficking and BDNF signaling.

## Results

### SORCS2 alternative splicing generates four variants with different ICDs

**Figure 1:**
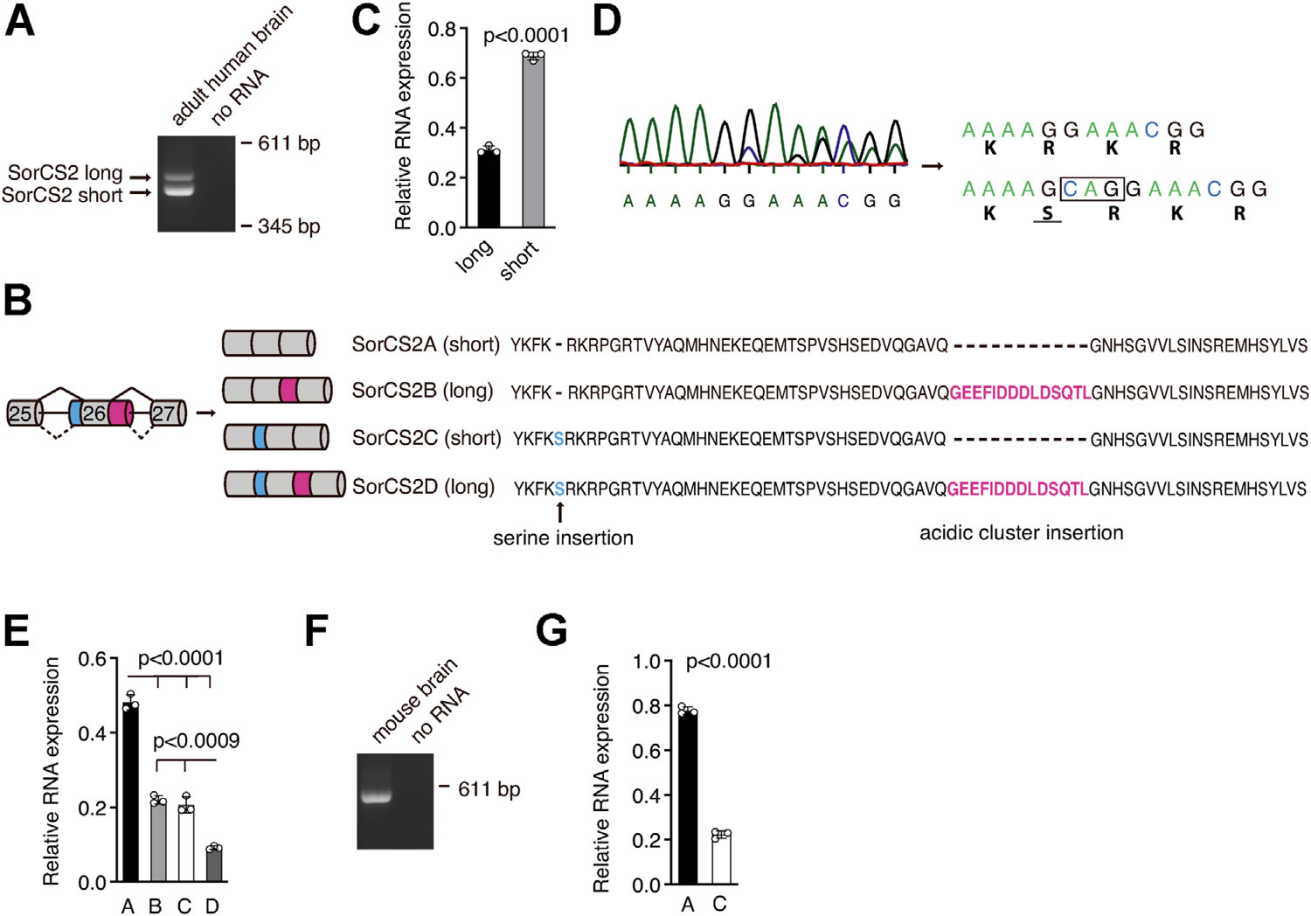
**Alternative splicing generates four mRNA isoforms of human SorCS2 in adult brain tissue.***A*, RT-PCR amplification of mRNA encoding the C-terminal end of SorCS2 reveals two bands that is, SorCS2 long and short in human brain tissue. *B*, overview of alternative splicing pattern of human *SORCS2* pre-mRNA and deduced amino acid sequences of the intracellular domain. Numbers indicate exon numbers. *C*, densitometric quantification of *A*. *D*, sequencing of SorCS2 long and short revealed insertion of three nucleotides encoding a serine residue. Figure *D* shows a representative mixed sequencing profile of SorCS2 short. *E*, the mixed sequencing profiles allowed estimation of the ratio between *SORCS2* transcripts with and without the three-nucleotide insertion. *F*, RT-PCR amplification of mRNA 3′-end encoding mouse SorCS2 cytosolic domain. *G*, DNA sequencing of mouse *Sorcs2* RT-PCR product revealed the existence of two transcripts, one of which contained an insertion of three nucleotides encoding a serine residue. Data in each graph represent mean ± SD of three technical replicates. A *t* test was used to compare groups.

We searched the National Center for Biotechnology Information Expressed Sequence Tag database and identified several human *SORCS2* clones displaying exon–exon junction variation. The putative translational products indicated the existence of four ICDs. To assess whether human SorCS2 mRNA is indeed alternatively spliced, human adult brain RNA was subjected to RT-PCR, amplifying fragments encoding SorCS2 ICD. Two DNA fragments denoted SorCS2 long and SorCS2 short, respectively, were amplified (Fig. 1*A*). DNA sequencing confirmed the identity of the Expressed Sequence Tag clones. The variants were denoted as SorCS2A, SorCS2B, SorCS2C, and SorCS2D (Fig. 1*B*). The differential 5′ splice site in intron 26 introduces an acidic cluster of 14 amino acids in variant SorCS2A, giving rise to SorCS2B. The differential 3′splice site in intron 25 introduces a serine residue at position 1104 (S1104) in variant SorCS2A giving rise to SorCS2C, whereas use of both alternative splice sites gives rise to SorCS2D (Fig. 1*B*). The acidic cluster motif within the long variants B and D is found in 31.2% of SorCS2 transcripts from human adult brain (Fig. 1*C*). The mixed complementary DNA (cDNA) sequencing profiles of the amplified long and short RT-PCR fragments allowed quantification of the ratio between SorCS2 transcripts with or without the three-nucleotide insertion encoding S1104 (Fig. 1*D*). This further enabled us to estimate the relative distribution of the four *SORCS2* mRNA isoforms in whole brain tissue. Expression of *SORCS2A* mRNA prevailed, while *SORCS2D* exhibited the lowest level of expression (Fig. 1*E*). Interestingly, only two *Sorcs2* transcripts were detectable in mouse brain, one of which contains an alternatively spliced insertion of three nucleotides in analogy to the human *SORCS2C* isoform (Fig. 1*F*). Hence, SorCS2 variants expressed in mouse brain do not contain the acidic cluster motif. Like human brain, expression of the *Sorcs2A* variant prevailed in mouse brain tissue being more than 3-fold higher than that of the mouse *Sorcs2C* variant (Fig. 1*G*).

### SorCS2 splicing is differentially regulated during development and in tissues

**Figure 2:**
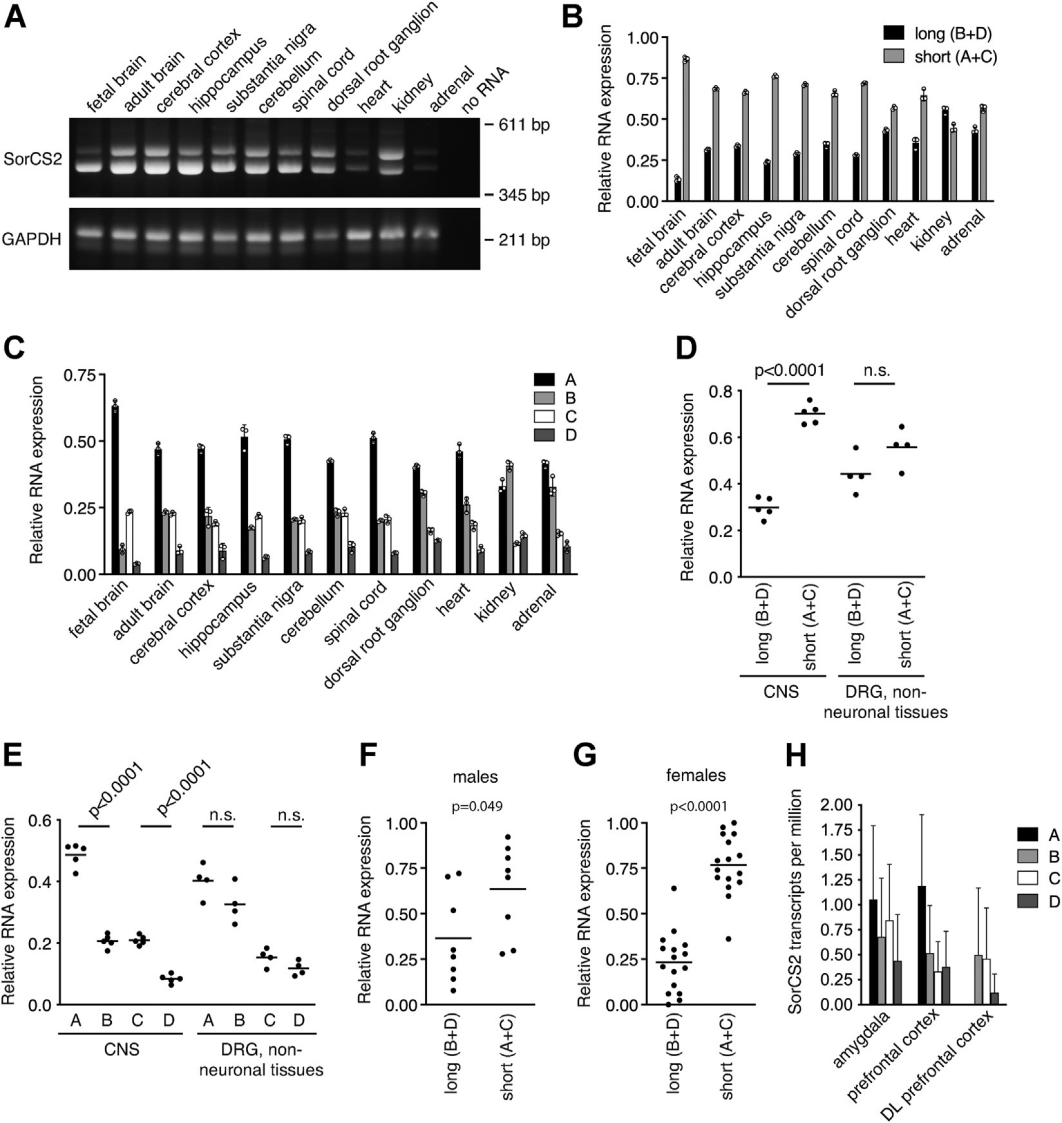
***SORCS2* mRNA isoforms are differentially expressed in a time- and tissue-specific manner.***A*, RT-PCR using human RNA preparations showed that SorCS2 short is the major mRNA variant expressed by various tissues within CNS. Dorsal root ganglia and the non-neuronal tissues heart, kidney, and adrenal gland showed a more even distribution of SorCS2 long and short. *B*, quantification of *A*. *C*, quantification of relative *SORCS2* mRNA isoforms by sequencing of SorCS2 long and short from *A*. Data from *B* and *C* represent three technical replicates. *D* and *E*, data from *B* and *C* were summarized, suggesting that *SORCS2* pre-mRNA is subjected to tissue-specific splicing. CNS tissues: cerebral cortex, hippocampus, substantia nigra, cerebellum, and spinal cord; nonneuronal tissues: heart, adrenal gland, and kidney. *F* and *G*, relative mRNA expression of SorCS2 long and short in peripheral blood from human adult males (*F*) and females (*G*). *H*, expression of *SORCS2* mRNA isoforms in amygdala (n = 21), prefrontal cortex (n = 8), and dorsolateral (DL) prefrontal cortex (n = 19). Data in *D*, *E*, *F*, and *G* are shown as mean and *B*, *C*, and *H* as mean ± SD. Comparisons of data between groups were performed with a *t* test.

We next sought to determine the relative distribution of human SorCS2 splice variants in different tissues. Cerebral cortex, hippocampus, substantia nigra, cerebellum, and spinal cord all showed expression of both SorCS2 long (SorCS2B+D) and short (SorCS2A+C) mRNA (Fig. 2, *A*, *B*, and *D*). The relative expression of *SORCS2A* was 2- to 3-fold higher than *SORCS2B* and *SORCS2C*. *SorCS2D* expression was further 2- to 3-fold reduced compared to *SORCS2B* and *SORCS2C* (Fig. 2, *C* and *E*). In fetal brain, an even higher relative expression of *SORCS2A* than *SORCS2B* and *SORCS2D* was observed, suggesting that SorCS2 splicing is developmentally regulated. In contrast to the CNS, dorsal root ganglia, heart, kidney, and adrenal gland expressed *SORCS2A* and *SORCS2B* at a more similar level. *SORCS2C* and *SORCS2D* are also found at roughly similar levels, however the relative expression is approximately 2- to 3-fold reduced compared to *SORCS2A* and *SORCS2B* (Fig. 2, *C* and *E*). To study how SorCS2 splicing varies among individuals, we performed RT-PCR on RNA extracted from blood samples of eight men and 16 women. Similar to neuronal tissues, the relative expression of SorCS2 short (SorCS2 A+C) prevails in both men and women (Fig. 2, *F* and *G*). To study SorCS2 splicing variation in CNS tissues, we studied RNA sequencing data from The Sequence Read Archive. These data do not allow for statistical analysis; however, it is clear that the relative expression of splice variants in amygdala and prefrontal cortex is similar to that observed in other CNS tissues (Fig. 2*H*). Surprisingly, there was a complete lack of *SORCS2A* expression in the dorsolateral prefrontal cortex.

### Cellular stress affects SorCS2 splicing

**Figure 3:**
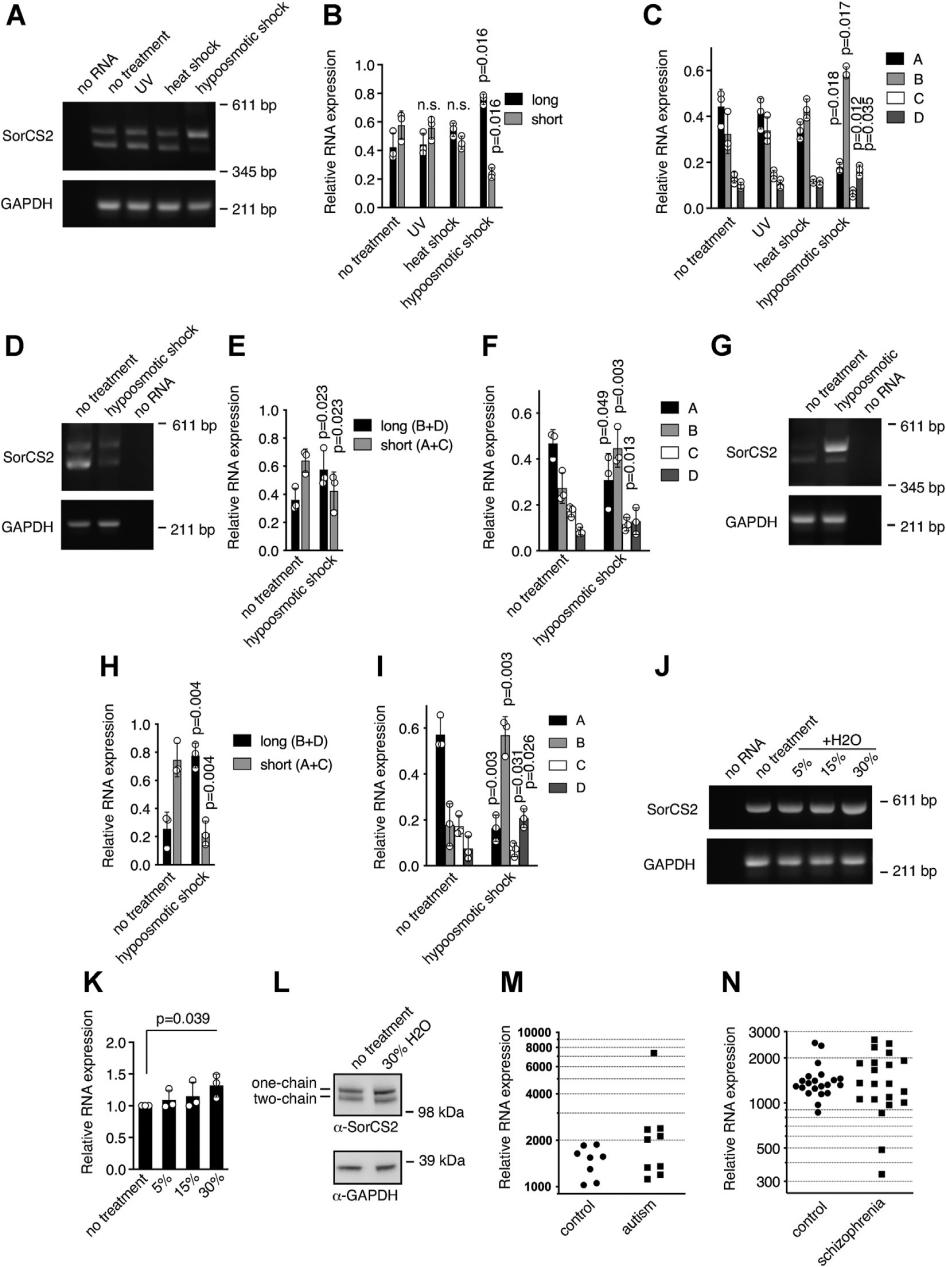
**Cellular stress regulates SorCS2 alternative splicing.***A*, RT-PCR of *SORCS2* mRNA from HEK293 cells subjected to cellular stress as indicated. Cellular stress increases the expression of SorCS2 long and decreases the expression of SorCS2 short. *B*, densitometric quantification of *A*. *C*, quantification of *SORCS2* mRNA isoforms in HEK293 cells subjected to stress. *D* and *G*, RT-PCR of *SORCS2* mRNA from HepG2 (*D*) or SH-SY5Y (*G*) cells were subjected to hypoosmotic conditions. *E* and *H*, densitometric quantification of *D* and *G*, respectively. *F* and *I*, quantification of *SORCS2A*, *SORCS2B*, *SORCS2C*, and *SORCS2D* mRNA in HepG2 (*F*) and SH-SY5Y (*I*) cells were subjected to hypoosmotic stress. *J*, six DIV hippocampal cultures were treated with hypoosmotic medium as indicated for 24 h and *Sorcs2* mRNA was assessed by RT-PCR. *K*, densitometric quantification of *J*. *L*, representative Western blot showing increased SorCS2 levels after hypoosmotic shock. *M* and *N*, normalized expression of *SORCS2* transcripts in prefrontal cortex of autism patients (*M*) and amygdala of schizophrenia patients (*N*) compared to healthy control groups. Data in *B*, *C*, *E*, *F*, *H*, *I*, and *K* represents mean ± SD of three experiments. Relative stress-induced mRNA expression of *SORCS2* isoforms was compared to the corresponding isoform from nontreated cells using a *t* test. Variances in *SorCS2* mRNA levels in patients suffering from autism or schizophrenia and control groups were compared using an F-test. HEK, human embryonic kidney.

The differential SorCS2 splicing in tissues and during development prompted us to address whether SorCS2 splicing is also regulated by different cellular conditions. We subjected human embryonic kidney (HEK293) cells to various stress conditions and assessed changes in SorCS2 splicing pattern by RT-PCR. UV radiation of cells did not affect the ratio between SorCS2 long and short. Heat shocking of cells at 42 °C for 2 h increased although not significantly the relative amount of SorCS2 long relative to SorCS2 short (Fig. 3, *A* and *B*). However, the splicing pattern was significantly changed when subjecting cells to hypoosmotic medium for 24 h, leading to approximately 2-fold increase in *SORCS2B* and *SORCS2D* transcripts (Fig. 3*C*). Similar findings were found for the liver cell line HepG2 and the neuronal cell line SH-SY5Y when subjected to hypoosmotic shock (Fig. 3, *D*–*I*). Strikingly, although untreated HEK293, HepG2, and SH-SY5Y cells possess different *SORCS2* mRNA isoform expression profiles under normal culture conditions, hypoosmotic stress resulted in a strikingly similar expression profile of *SORCS2* isoforms in all three cell types. Interestingly, hypoosmotic stress had no effect on *Sorcs2* splicing in mouse hippocampal neurons. However, there was a clear increase in total *Sorcs2* transcripts as well as translated product (Fig. 3, *J*–*L*).

To study how pathological conditions might affect *SORCS2* expression, we compared CNS levels of total *SORCS2* transcripts in patients suffering from autism or schizophrenia with healthy control groups using data from The Sequence Read Archive. In autism patients, mean *SORCS2* expression was non-significantly increased by ∼60% in prefrontal cortex. Interestingly, *SORCS2* expression levels spanned a much greater range for autism patients than the control group (*p* = 0.00011) (Fig. 3*M*). Likewise, the variation was markedly increased in amygdala in patients suffering from schizophrenia compared to healthy individuals (*p* = 0.047), suggesting that *SORCS2* brain expression could be perturbed in a general manner in patients suffering from psychiatric disorders (Fig. 3*N*).

### Alternative splicing and posttranslational processing generate eight SorCS2 isoforms

**Figure 4:**
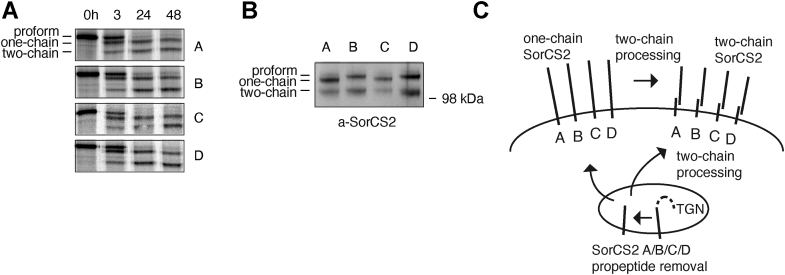
**Posttranslational processing of SorCS2 splice variants generates a total of eight isoforms.***A*, to follow receptor processing, HEK293 cells were transfected with *SORCS2* isoform cDNA, pulsed with ^35^S and chased up to 48 h. *B*, representative Western blot showing that distribution of SorCS2 processing variants at steady state is similar between SorCS2 isoforms in HEK293 cells. *C*, schematic overview of predicted SorCS2 isoforms as a result of alternative splicing and receptor processing. The proform of SorCS2 is converted into one-chain receptor within *trans*-Golgi compartments (TGN). One-chain SorCS2 may be processed into two-chain receptor either during trafficking through the secretory pathway or on the cell surface. HEK, human embryonic kidney.

*SORCS2* mRNA is translated into a proprotein containing a short propeptide, which is proteolytically processed to generate a single-chain receptor, which may eventually be processed into two-chain SorCS2 (9). So far, all biochemical and functional studies using transfected cells, including receptor-processing experiments, have been performed using cDNA encoding the SorCS2A isoform (9, 10, 11, 20). To gain insight into the processing of the additional SorCS2 splice variants, we cloned SorCS2B, SorCS2BC, and SorCS2BD and followed receptor maturation by ^35^S-pulse chase analysis in transfected HEK293 cells. All four receptor variants were processed from their proform into single- and two-chain receptors in a similar manner (Fig. 4*A*). We also assessed steady state distribution of pro-, one-, and two-chain receptors for each of the splice variants by Western blotting using HEK293 cells. All receptor-processing variants are present at similar levels with one-chain receptor being the most abundant species, followed by two-chain SorCS2 (Fig. 4*B*). Thus, it can be predicted that posttranslational proteolytic processing gives rise to a total of eight SorCS2 isoforms (Fig. 4*C*).

### The presence of Ser1104 inhibits SorCS2 neurotrophic activity

**Figure 5:**
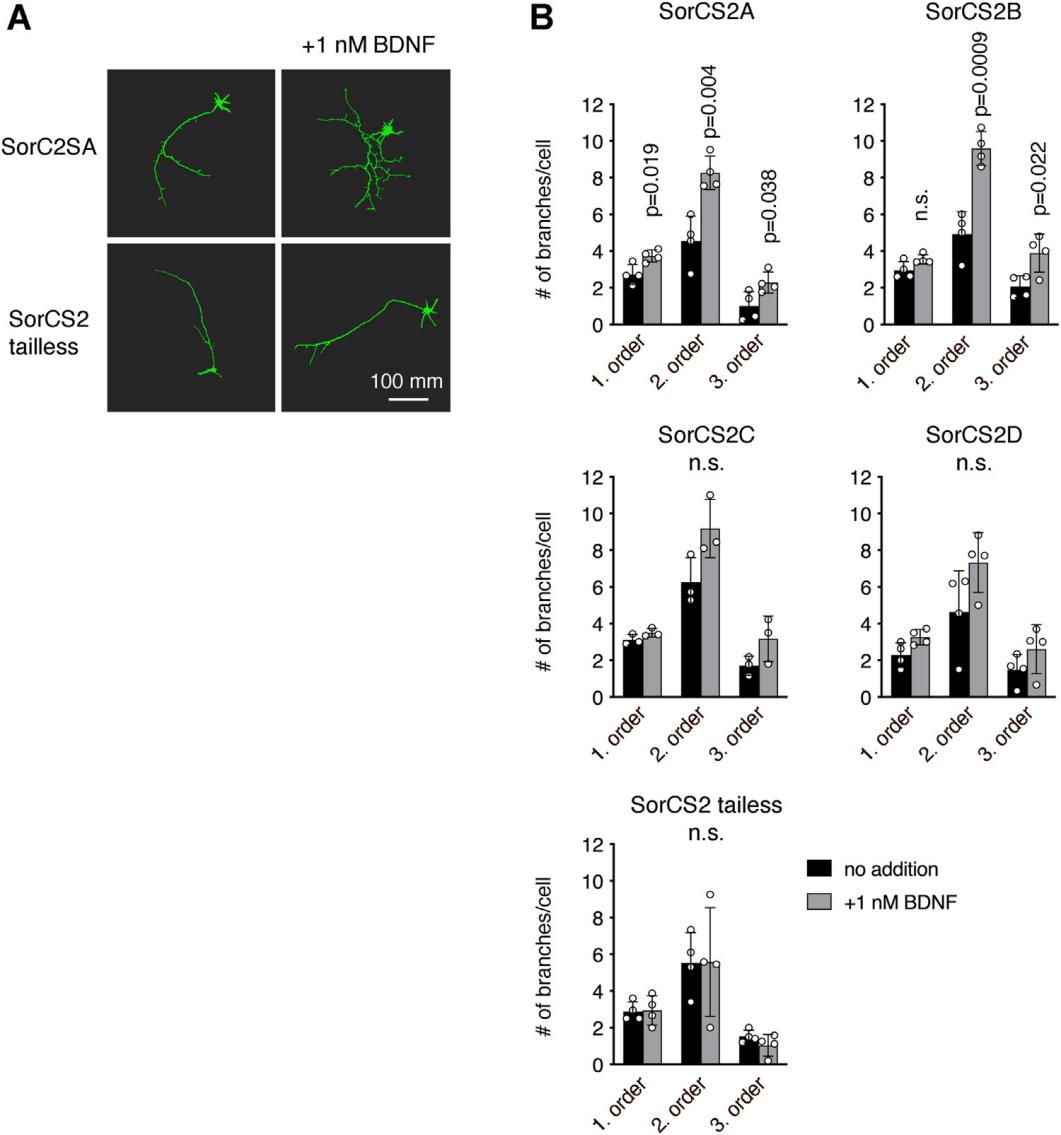
**Insertion of S1104 in SorCS2 hampers BDNF-induced neuronal branching.** SorCS2 KO hippocampal neurons were cotransfected with GFP and indicated SorCS2 constructs and treated with 1 nM BDNF for 72 h. *A*, representative images of neurons transfected with SorCS2A or SorCS2 tailless in the presence or absence of BDNF. Images have been reconstructed using Imaris software (https://imaris.oxinst.com/). *B*, quantification of neuronal branching for neurons transfected with SorCS2A, SorCS2B, SorCS2C, or SorCS2D in the presence or absence of BDNF. Data in graphs represent mean ± SD of three or four experiments. Treatment responses in different groups were compared by two-way ANOVA. If the analysis of variance revealed significant differences, a *t* test was carried out to elucidate the pattern of differences.

The SorCS2 ICD is required for BDNF-induced branching of hippocampal neurons (11). We therefore assessed if alternative splicing of SorCS2 affects this process. Thus, primary cultures of SorCS2 KO mouse hippocampal neurons were transfected with cDNA encoding the individual SorCS2 splice variants and cultured in the absence or presence of 1 nM BDNF for 72 h. As previously reported, transfection with a SorCS2 truncation variant lacking the entire ICD (SorCS2 tailless) failed to rescue BDNF-induced branching of SorCS2 KO neurons (11). In contrast, transfection with SorCS2A and SorCS2B significantly rescued BDNF-induced branching (Fig. 5*B*). However, no significant rescue was observed with SorCS2C and SorCS2D, suggesting that insertion of a serine residue at position 1104 in SorCS2 ICD compromises BDNF-signaling activity.

### SorCS2 alternative splicing affects receptor trafficking and interactions with sorting APs

**Figure 6:**
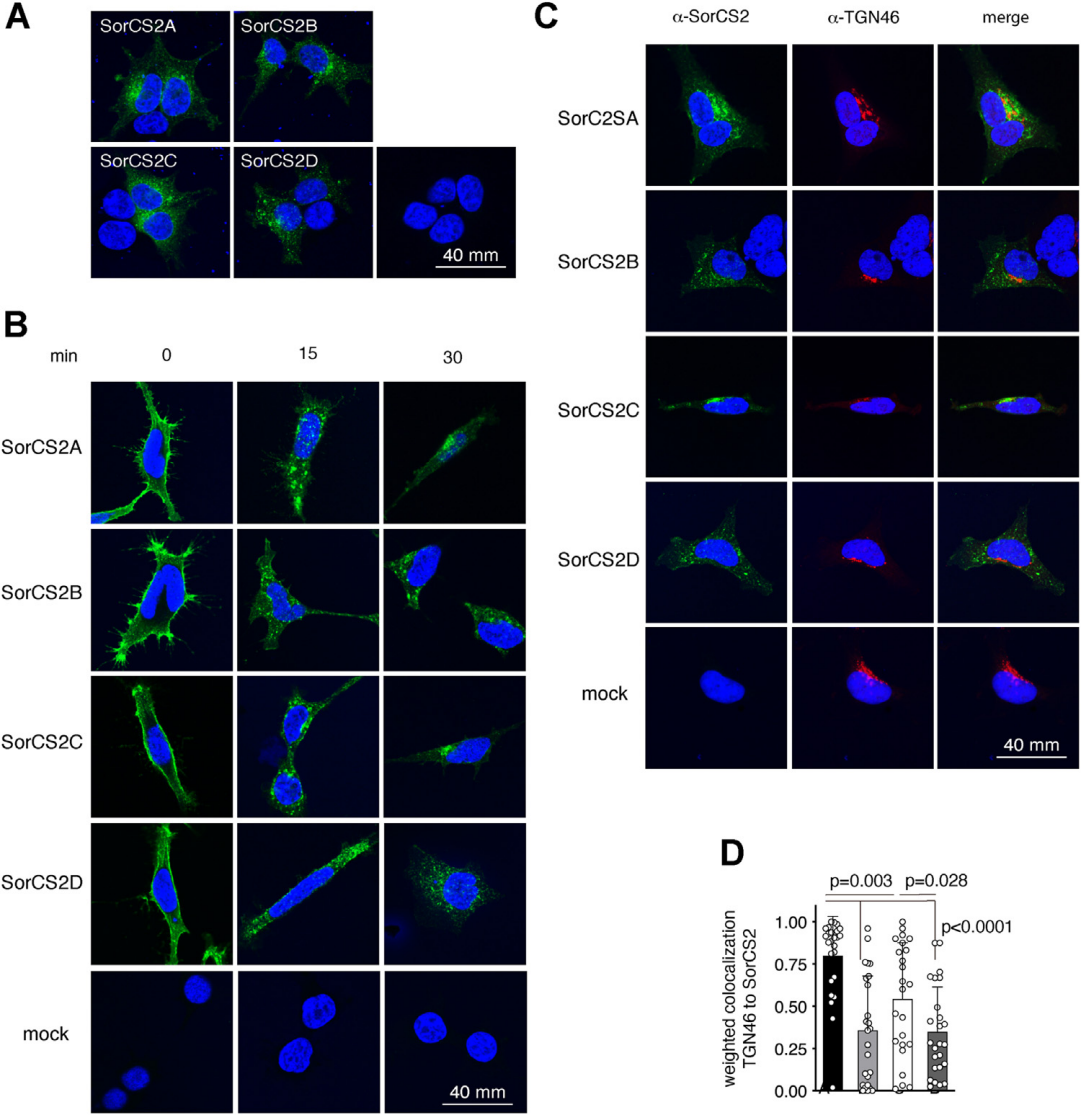
**SorCS2 splice variants display different trafficking.***A*, SorCS2-immunostained HEK293 cells transfected with SorCS2A and SorCS2C show a more paranuclear staining pattern compared to cells transfected with SorCS2B and SorCS2D for which staining is distributed in vesicular structures throughout the cell. *B*, surface SorCS2 was labeled with an antibody directed against its extracellular domain on ice. Trafficking was subsequently monitored by incubation at 37 °C, followed by fixation, permeabilization, and staining with a secondary antibody at indicated time points. *C*, after 30 min, more SorCS2A and SorCS2C accumulate in paranuclear structures positive for the *trans*-Golgi marker TGN46 compared to SorCS2B, and D. *D*, quantification of *C*. The data in the graph is shown as mean ± SD and comparisons between groups conducted using a *t* test.

Intracellular sorting of SorCS2 is governed by its ICD (9), and we therefore assessed the subcellular localization of each of the splice isoforms by immunostaining of transfected HEK293 cells. We found that all four splice isoforms were localized in vesicle-like structures and at the cell surface. However, the staining pattern of SorCS2A and SorCS2C lacking the acidic cluster motif was more paranuclear localized compared to SorCS2B and SorCS2D (Fig. 6*A*). We therefore monitored trafficking of SorCS2 isoforms by labeling surface-localized SorCS2 on ice with an antibody directed against the extracellular domain, followed by incubation at 37 °C for defined time intervals, whereafter cells were fixed and stained with secondary antibodies. At 15 min, all four receptor variants were mainly found in smaller vesicles. However, at 30 min, SorCS2A and SorCS2C mainly localized to paranuclear compartments in contrast to SorCS2B and SorCS2D, which were still observed more distant from the nucleus (Fig. 6*B*). Internalized SorCS2A and SorCS2C showed partial overlap with the *trans*-Golgi marker TGN46, suggesting retrograde transport to the *trans*-Golgi network (Fig. 6, *C* and *D*).

**Figure 7:**
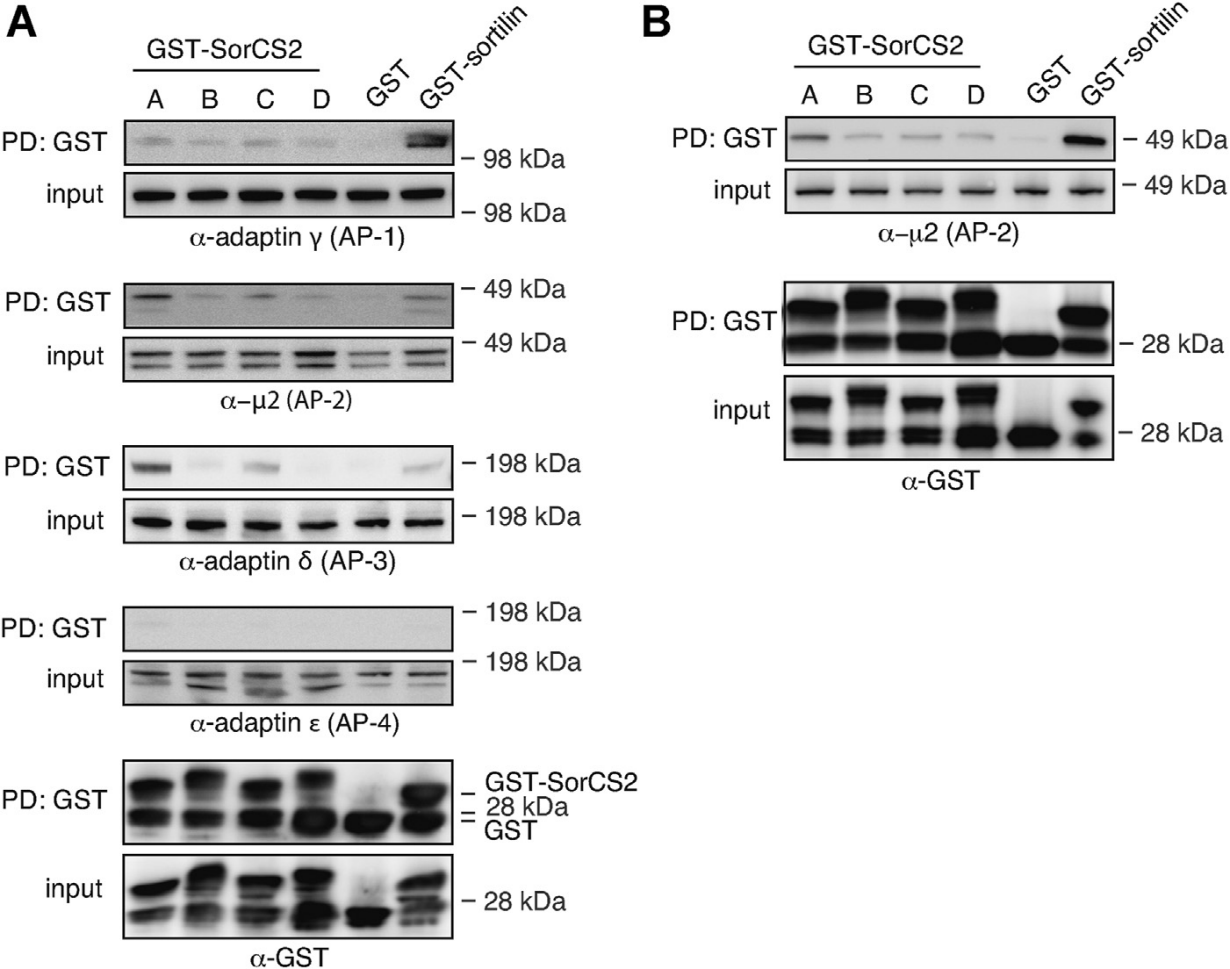
**Clathrin adaptor proteins preferentially interacts with SorCS2A and SorCS2C.** Representative Western blots (n = 3) of pull downs using GST-fusion proteins encompassing SorCS2 or sortilin ICD, showing coprecipitation of AP complexes from HEK293 cell lysate (*A*) or human brain homogenate (*B*). Please note that free GST protein is generated as a consequence of partial degradation and commonly observed for GST-fusion proteins. AP, adaptor protein; GST, glutathione-*S*-transferase; HEK. human embryonic kidney; ICD, ICD, intracellular domain; PD, pull down.

The SorCS2 ICD contains a tyrosine-based internalization and sorting motif YAQM conforming to the consensus sequence YXXØ (where X is any amino acid and Ø is a hydrophobic, bulky residue) (Fig. 1*D*). Clathrin AP complexes are known to bind such motifs in other receptors, and thereby mediate their sorting between the cell surface, endosomes, and TGN (21, 22). Sorting of the Vps10p domain receptor sortilin relies on AP-1 (23) whereas sorting of SorLA depends on both AP-1 and AP-2 (24), but so far, the interaction between SorCS2 and APs has not been studied. We therefore generated fusion proteins between SorCS2 ICD and glutathione-*S*-transferase (GST) and assessed pull down of endogenous AP complexes from HEK293 cell lysate. We used the ICD of sortilin fused to GST as positive control and confirmed the interaction with AP-1 (23) but also observed that sortilin binds AP-2 and AP-3, which has not previously been described (Fig. 7*A*). Interestingly, SorCS2A ICD conferred the most robust pull down of AP-1, AP-2, and AP-3, followed by SorCS2C ICD. SorCS2B and SorCS2D pulled down AP-1 and AP-2 to a lower extent but showed no interaction with AP-3, suggesting that the presence of the acidic cluster in SorCS2B and SorCS2D potentially obstructs the interaction with AP complexes. The adaptin ε subunit from AP-4 was not pulled down by any of the SorCS2 ICD fusion proteins. The interaction between SorCS2 ICD and AP-2 was confirmed using human brain homogenate (Fig. 7*B*), however we were not able to detect human AP-1 and AP-3 in neither pull downs nor homogenates from human brain tissue by Western blotting.

### SorCS2 alternative splicing regulates interactions with motor proteins kinesin and dynein

**Figure 8:**
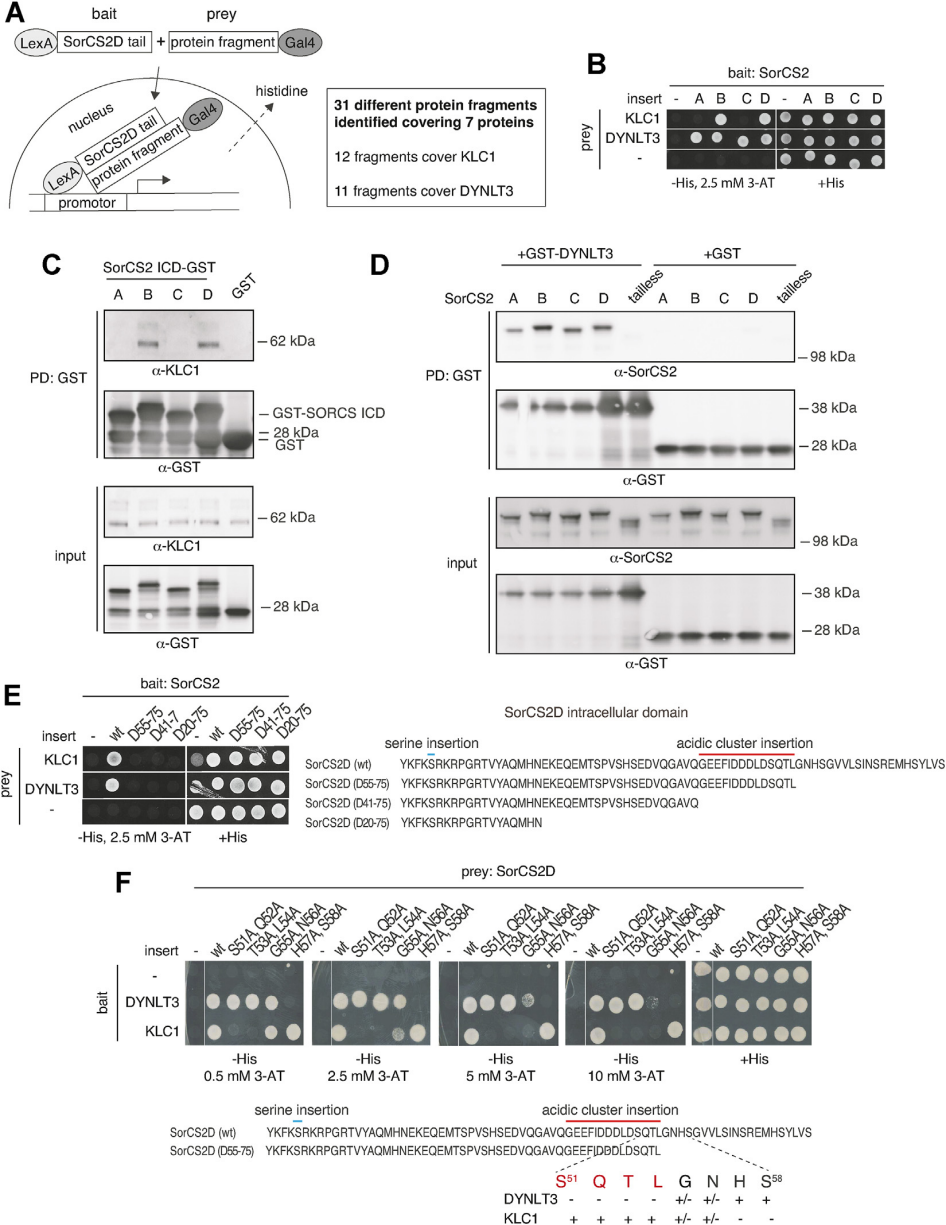
**SorCS2 variants selectively interacts with KLC1 and DYNLT3.***A*, illustration of yeast two-hybrid screen used for identification of SorCS2D ICD–binding partners. SorCS2D ICD fused to the DNA-binding protein LexA constitutes the bait, which binds upstream of a histidine reporter gene. Protein fragments from an adult human brain library fused to the activation domain (AD) of the Gal4 transcription factor is referred to as the prey. Only a physical interaction between bait and prey allows transcription of the histidine reporter gene and growth in the absence of histidine. Aside from fragments covering KLC1 and DYNLT3, the remaining eight fragments covering five proteins reached a score of 3 or less. *B*, representative yeast two-hybrid assay showing an interaction between SorCS2B and SorCS2D ICD with KLC1 and between ICD of all four isoforms with DYNLT3. *C*, SorCS2B and SorCS2D ICD fused to GST pull-down endogenous KLC1 from HEK293 cell lysate. *D*, DYNLT3-GST pulls down all SorCS2 isoforms from transfected HEK293 cell lysate. *E*, mapping by yeast two-hybrid shows that deletion of the C-terminal 20 amino acids of SorCS2D ICD compromises interactions with KLC1 and DYNLT3. *F*, representative yeast two-hybrid assay showing that substitutions with alanine mainly within the acidic cluster of SorCS2D ICD abrogates interactions with KLC1. In contrast, only substitutions outside the acidic cluster abrogates interactions with DYNLT3. DYNLT3, dynein light chain Tctex-type 3; ICD, intracellular domain; KLC, kinesin light chain; PD, pull down.

To search for additional sorting APs contributing to SorCS2 isoform specific trafficking, we performed genome-wide yeast two-hybrid screening at Hybrigenics using SorCS2D ICD as bait and screened a human adult brain prey library containing 10 million primary clones. Thirty one protein fragments representing a total of seven cytosolic proteins were found to interact with SorCS2D ICD. Each hit was scored on a scale from 1 to 6 based on local and global technical parameters, with six representing very high confidence in the interaction. Twelve fragments, all of which obtained a score of 6, covered kinesin KLC1 a subunit of the motor protein kinesin responsible for cargo transport along microtubules toward the plus end (anterograde transport). Eleven fragments, again all of which obtained the highest score, covered DYNLT3 a subunit of the motor protein dynein, which moves cargo along microtubules toward the minus end (retrograde transport) (Fig. 8*A*). We subsequently assessed KLC1 and DYNLT3 binding to all four SorCS2 isoforms by yeast two-hybrid assay using plasmids obtained from the global yeast-two hybrid screen. We confirmed an interaction between SorCS2D ICD and KLC1 but also SorCS2B ICD–bound KLC1. We also confirmed the interaction between SorCS2D ICD and DYNLT3 and in addition found that the remaining three SorCS2 ICD isoforms also interact with DYNLT3 (Fig. 8*B*). We corroborated these findings by pull-down experiments using GST-SorCS2 ICD constructs to pull down endogenous KLC1 from HEK293 cell lysate (Fig. 8*C*) and GST-DYNLT3 to pull down SorCS2 isoforms from transfected HEK293 cell lysate (Fig. 8*D*). In order to further explore the specificity of SorCS2 splice variants in terms of motor protein adaptor binding, we sought to define the epitope of SorCS2 ICD responsible for the interaction with KLC1 and DYNLT3. To this end, we performed yeast two-hybrid assay using C terminally truncated variants of SorCS2D ICD as bait. Removal of the most C-terminal 20 amino acids fully abrogated interactions with both KLC1 and DYNLT3, suggesting that amino acids within this sequence and/or within the most C-terminal part of the acidic cluster motif contribute to KLC1 and DYNLT3 binding (Fig. 8*E*). To fine map the binding epitope of SorCS2D, we performed alanine scanning of SorCS2D C terminus, including part of the acidic cluster. Pairwise alanine substitutions of Ser^51^-Gln^52^ and Thr^53^-Leu^54^ located within the acidic cluster and to some extent Gly^55^-Asn^56^ located outside the acidic cluster (numbered according to the cytosolic sequence of SorCS2D ICD) abrogated KLC1 binding (Fig. 8*F*). In contrast, only alanine substitutions of amino acids located outside the acidic cluster (Gly^55^-Asn^56^ and His^57^-Ser^58^) inhibited DYNLT3 binding. Hence, KLC1 and DYNLT3 bind to distinct yet partly overlapping epitopes with KLC1 binding to the acidic cluster motif.

Thus, the differential trafficking properties of the SorCS2 splice isoforms may in part be explained by isoform-dependent preference for KLC1 and DYNLT3.

**Figure 9:**
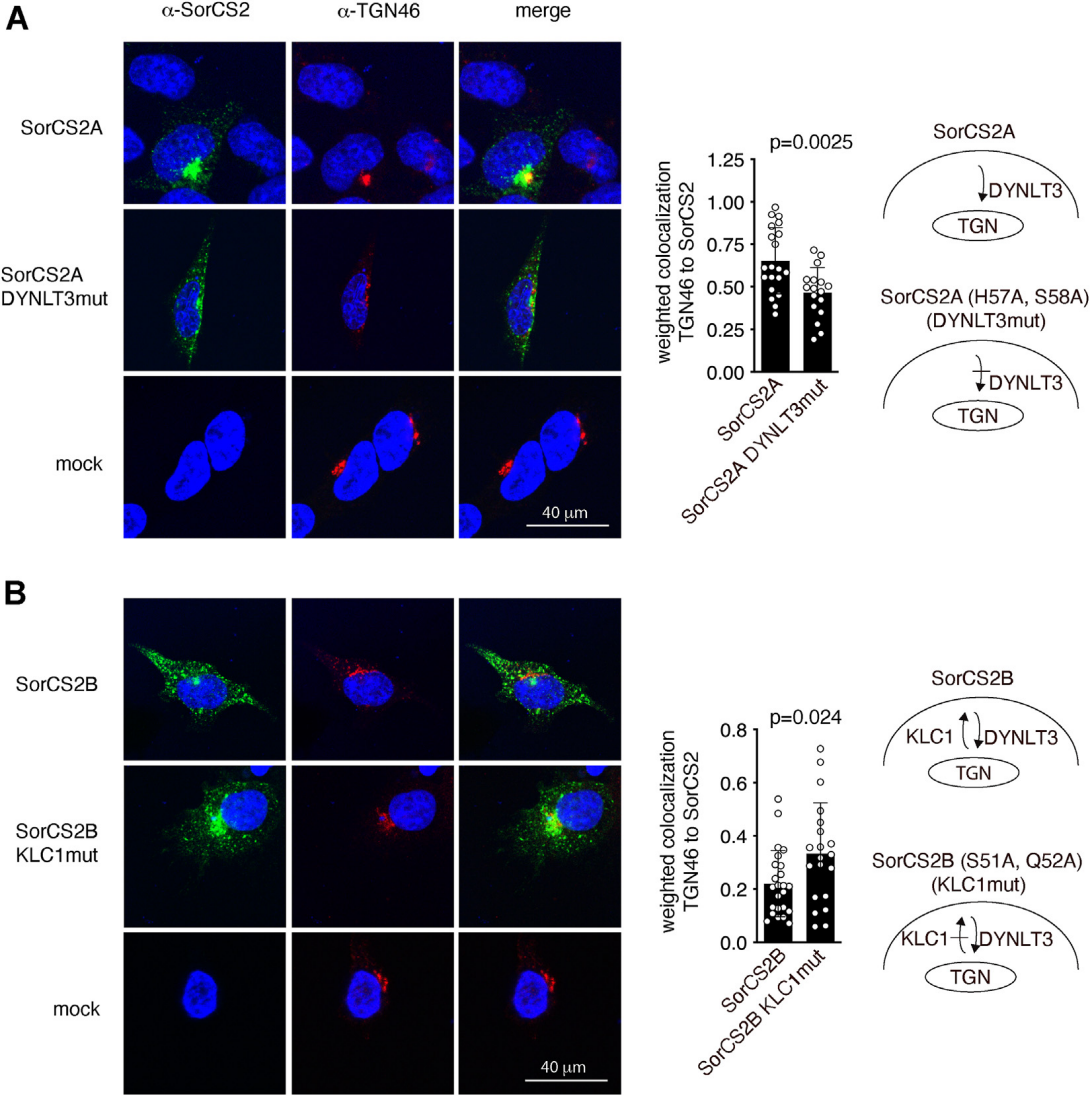
**Interactions with KLC1 and DYNLT3 regulate SorCS2 isoform anterograde and retrograde trafficking.** Trafficking of cell surface SorCS2 variants were followed by antibody uptake experiments. Data represent SorCS2 subcellular distribution after 30 min at 37 °C. *A*, mutation of the DYNLT3-binding site of SorCS2A reduces its accumulation in TGN46-positive paranuclear compartments. *B*, mutation of the KLC1-binding site of SorCS2B increases its accumulation in TGN46-positive paranuclear compartments. The data in the graphs are shown as mean ± SD and comparisons between groups conducted using a *t* test. DYNLT3, dynein light chain Tctex-type 3; KLC, kinesin light chain.

To study the influence of DYNLT3 on SorCS2 trafficking, we mutated the DYNLT3-binding site of SorCS2A by substituting His^57^ and Ser^58^ with alanine (SorCS2A DYNLT3mut). We then monitored trafficking of DYNLT3mut and wt SorCS2A by antibody uptake studies in transfected HEK293 cells. After 30 min, SorCS2A DYNLT3mut was mainly found in vesicle-like structures evenly distributed throughout the cytoplasm compared to wt SorCS2A, which showed a more paranuclear staining pattern (Fig. 9*A*). Hence, colocalization between SorCS2A DYNLT3mut and TGN46 was significantly reduced compared to that for wt SorCS2A, consistent with decreased retrograde transport of SorCS2A DYNLT3mut. We next assessed the involvement of KLC1 in SorCS2 trafficking by mutating part of the KLC1-binding site of SorCS2B by substituting Ser^51^ and Gln^52^ with alanine (SorCS2B KLC1mut). Whereas wt SorCS2B was mainly found in vesicular structures distributed throughout the cytoplasm after 30 min, SorCS2B KLC1mut staining was more paranuclear (Fig. 9*B*). The results are consistent with reduced anterograde transport of SorCS2B KLC1mut, shifting its subcellular distribution from cytoplasmic to paranuclear. Hence, our findings suggest that alternative splicing regulates SorCS2 anterograde and retrograde transport through interactions with kinesin and dynein motor proteins.

## Discussion

Alternative splicing generates unique protein isoforms to carry out specialized functions at specific time points in distinct tissues (1, 2) down to the level of individual cells (25). Most often, alternative splicing events affect intrinsically disordered amino acid sequences or sequences located on the protein surface (26, 27). Therefore, alternative splicing usually does not influence the 3D structure of spliced proteins but rather introduce small-sized changes with new protein–protein interaction capabilities (26, 27). The second most common form of alternative splicing after exon inclusion/skipping involves usage of an alternative splice site at the 5′ or 3′ end of introns when exons are assembled. We here report the identification of four splice variants of human SorCS2 as a result of alternative splice site selection at the 5′ and/or 3′ end in two consecutive introns. This gives rise to insertion of a serine residue at position 1104 and/or an acidic cluster motif inserted between amino acids 1139 and 1140 within the ICD of SorCS2A. As each splice variant can be further posttranslationally modified through proteolytic processing into both one- and two-chain SorCS2, a total number of eight receptor variants are predicted to exist. Interestingly, the expression profiles of SorCS2 splice variants display a dynamic variation in both a developmental and tissue-dependent manner, suggesting distinct functional properties. As such, the SorCS2 variants possess differential trafficking properties. Hence, upon endocytosis SorCS2A and SorCS2C rapidly accumulate in paranuclear compartments in contrast to SorCS2B and SorCS2D, which are clustered in vesicular structures more distant from the nucleus. Consistent with this, we find that the clathrin-associated APs AP-1, AP-2, and AP-3 preferentially bind SorCS2A and SorCS2C. In particular, AP-3 exclusively interacted with these two variants. The individual AP complexes are critical for specific sorting of cargo between the different subcellular compartments of polarized cells (28). For example, AP-1 is known to be essential for somatodendritic sorting of neurotransmitter receptors (29). In line with this, mouse SorCS2 is localized in dendrites of hippocampal neurons (11). Thus, it will be of interest to study how AP-1, AP-2, and AP-3 complexes influence the subcellular and in particular dendritic localization of SorCS2 variants in neurons.

SorCS2 is involved in proBDNF/BDNF activity and influences cell survival, axon guidance, and synaptic plasticity (9, 10, 11, 20). Interestingly, BDNF-induced changes in synaptic strength and neuronal complexity are dependent on SorCS2 ICD (11). We here find that only transfection with constructs encoding SorCS2A and SorCS2B is able to significantly rescue BDNF-induced branching of SorCS2 KO hippocampal neurons. Thus, the presence of a serine at position 1104 appears to hamper downstream effects of BDNF signaling. In this light, it is interesting that a cell type–specific alternative splicing program has been demonstrated for hippocampal CA1 neurons (25), where SorCS2 is known to be critical for synaptic plasticity (11). Thus, it is tempting to speculate that a particular cell type or subcellular structure may experience completely different outcomes of BDNF and proBDNF signaling depending on which SorCS2 variant is present or absent in combination with p75^NTR^ or TrkB. Further studies are needed to elucidate the precise mechanism for how S1104 modulates BDNF signaling. However, we submitted the ICD sequences of SorCS2C and SorCS2D to NetPhos 3.1 (https://services.healthtech.dtu.dk/services/NetPhos-3.1/) (30, 31), a bioinformatics tool, that uses neural network–based algorithms to predict potential phosphorylation sites as well the responsible kinases. S1104 was predicted to be phosphorylated with a score of 0.98 out of 1, indicating high probability of phosphorylation as the threshold score for significant prediction is 0.5. PKC was predicted as the responsible kinase (score: 0.87 out of 1). This is interesting as BDNF-induced TrkB signaling depends on phospho lipase C gamma activity, which subsequently leads to activation of PKC, affecting both synaptic plasticity and neuronal branching (32, 33). Hence, it is tempting to speculate that phosphorylation of S1104 in SorCS2C-D variants as consequence of TrkB-phospho lipase C gamma activity shapes the neuronal response to BDNF.

Alternative splicing is often modulated by stress stimuli that promote synthesis of alternatively spliced protein isoforms as a strategy to cope with changing conditions and increase chance of survival (34). We here found that physical stressors affect SorCS2 splicing in three different human-derived cell lines and that especially hypoosmotic conditions induces upregulation of *SORCS2B* and *SORCS2D* transcripts both containing the acidic cluster motif. In the case of the neuronal SH-SY5Y cell line, hypoosmotic conditions did not only change the ratio between *SORCS2* mRNA isoforms but also induced a robust increase in the absolute numbers of *SORCS2* transcripts. The physiological implication of this observation is unclear but hypoosmotic extracellular conditions are a characteristic of intense neuronal activity within the retina (35). Under such circumstances, BDNF inhibits hypoosmotic cytotoxic cell swelling of Müller glial cells within the retina (35). Müller cells in turn protect surrounding neurons by releasing other neuroprotective cytokines (36). The BDNF signal is elicited through the truncated form of TrkB (TrkB-T), lacking the entire kinase domain (36, 37). Hence, it will be interesting to study SorCS2B and SorCS2D expression in Müller cells during hypoosmotic conditions and if these isoforms in conjunction with TrkB-T mediates the effect of BDNF.

Proper neuron functioning depends on axonal and dendritic transport of various membrane-bound organelles and other cellular cargoes along microtubules by motor proteins dynein and kinesin (38). By yeast two-hybrid and pull-down experiments, we found that all four SorCS2 variants interact with DYNLT3, whereas KLC1 specifically binds the acidic cluster motif in SorCS2B and SorCS2D. Dynein is a large 1.5 MDa multimeric protein complex composed of two heavy chains with motor function as well as a number of other components, including DYNLT3 all of which are present in two copies. These subunits or yet another protein complex dynactin confer dynein with cargo-binding specificity (39, 40). Cargo-binding specificity of kinesin superfamily proteins (KIFs) is achieved through structural diversity of the motor domains (39, 41). Hence, there is a total of 45 KIF genes in mammals, where the kinesin-1 family contains three members KIF5A, KIF5B, and KIF5C. Dimers of KIF5 isoforms constitutes the functional unit of the kinesin-1 members with two motor domains, a stalk region, and a tail, which binds KLC which associate with cargo (39, 41). Most often, dendritic and axonal transport of cargos such as membrane receptors *via* dynein and kinesin motor complexes require one or more adaptors in addition to DYNLT3 and KLC1 (40, 41). However, the interaction between SorCS2 isoforms and DYNLT3 and KLC1 appears to be direct as yeast two-hybrid used in our studies is based on protein–protein interaction of fused protein domains in the nucleus and therefore unlikely to involve cytosolic proteins that could bridge the interaction. In addition, we identified several clones covering different DYNLT3 and KLC1 sequences, whereas no other APs known to be involved in dynein and kinesin-based transport were found. Furthermore, direct membrane receptor transport *via* dynein light chain and KLC have previously been reported, suggesting that additional APs are not always needed for efficient membrane receptor transport (39, 40).

Several aspects of Trk receptor biology including BDNF-dependent TrkB signaling depends on long distance trafficking by dynein and kinesin motor proteins (42). As SorCS2 affects activity of mature BDNF and facilitates translocation of TrkB to postsynaptic densities (11), it can be speculated that such SorCS2 activities may also rely on dynein and kinesin motor activity. However, the presence of the acidic cluster in SorCS2B and SorCS2D did not affect BDNF-dependent neurite outgrowth in mouse cortical neurons (Fig. 5*B*), suggesting that BDNF-signaling properties of SorCS2 does not depend on kinesin motor activity at least not in this specific experimental paradigm. However, as mice do not express SorCS2 isoforms containing the acidic cluster, future studies should address the significance of the SorCS2 acidic cluster in BDNF signaling in a human context.

Besides translocating TrkB to postsynaptic densities, SorCS2 is also involved in anterograde dendritic transport of GluN2A (12). Previous work has demonstrated that TrkB is anterogradely trafficked by KIF5 *via* the adaptor complex CRMP2-SLC1-Rab27B and GluN2A by KIF3B (43, 44). Yet, trafficking of TrkB and GluN2A may be accomplished through alternative trafficking complexes as demonstrated for APP, which can engage directly with KIF5 but also *via* the adaptor JIP1 (45, 46). Hence, translocation of TrkB and GluN2A by SorCS2 *via* motor proteins could potentially represent an alternative trafficking mechanism of TrkB and GluN2A, allowing more refined regulation of local postsynaptic function when needed. Such regulation could be relevant during development and neuronal stress and be achieved through alternative splicing of SorCS2.

In conclusion, alternative splicing generates four SorCS2 variants differing in their ICD. All variants were found to interact with DYNLT3 through which anterograde trafficking of SorCS2 isoforms are apparently achieved. The SorCS2B and SorCS2D variants contain the insertion of an acidic cluster, which interferes with binding of sorting APs AP-1, AP-2, and AP-3, while mediating binding to KLC1, resulting in altered subcellular localization and trafficking. The SorCS2C and SorCS2D variants contain the insertion of a serine (S1104), which renders the variants ineffective in transducing BDNF activity. Tissue-specific posttranslational processing subsequently generates a total of eight SorCS2 isoforms with potentially different functional properties. Future studies should address the role of sorting and motor APs in regulating SorCS2 activity in neurons as well as the effect of cellular stress and neuronal activity on SorCS2 alternative splicing *in vivo* and its potential implications for signaling by pro and mature BDNF.

## Experimental procedures

### Constructs

SorCS2A (GenBank OQ616758) was inserted into pcDNA3.1/zeo (Invitrogen). SorCS2 splice variants were cloned by RT-PCR amplification of cDNA from human fetal brain RNA (Clontech). C-terminal fragments encoding the C terminal of human SorCS2B (GenBank OQ616759), SorCS2C (GenBank OQ616760), and SorCS2D (GenBank OQ616761) were produced. Fragments were substituted with the corresponding sequence in SorCS2A cloned into pcDNA3.1/zeo using an inherent and XhoI restriction site.

#### Expression of GST-fusion proteins in *Escherichia coli*

PCR fragments encoding the cytoplasmic domains of human SorCS2 isoforms were inserted into pGEX-4T-1 expression plasmid (GE Healthcare) using EcoRI and XhoI restriction sites (SorCS2A: Phe^1097^-Ser^1159^, SorCS2B: Phe^1097^-Ser^1173^, SorCS2C: Phe^1097^-Ser^1160^and SorCS2D: Phe^1097^-Ser^1174^). The cytoplasmic domain of sortilin: Lys^779^-Glu^831^ (NP_064356) was inserted into pGEX-4T-1 using a PCR-generated fragment and BamHI and EcoRI restriction sites. Fragments expressing DYNLT3 (NP_006511.1) and KLCI: F^150^-E^422^ (NP_006511.1) were derived from YTH prey plasmids and inserted into the pGEX4T1 vector. Constructs were expressed in the *E. coli* BL21 strain and affinity-purified on Glutathione-Sepharose beads (GE Healthcare) and dialyzed against PBS.

### Reverse transcription-polymerase chain reaction

RNA from cell lines were purified using the NucleoSpin gel and PCR clean-up kit from MACHEREY-NAGEL. RNA from human and mouse tissues were purchased from Clontech. Blood samples from human subjects were all part of the Danish PRISME study (47, 48) approved by the Danish Data Protection Agency (2006-41-7032) and the Danish Regional Ethics Committee (RRS 2006-1028 [2747-06]). All subjects were Caucasian of origin and gave written informed consent. The studies abide by the Declaration of Helsinki principles. PAXgene blood RNA tubes were used for collection and immediate stabilization of RNA (PreAnalytiX, Qiagen). RNA was extracted using standard procedures.

mRNA was converted to DNA and a fragment encoding the 3′ end of human SorCS2 including the cytosolic domain amplified with Titanium one-step RT-PCR kit (Clontech) using the primer pairs 5′-CAAGGAAGAGGAGCCTCTCGAGTGATA-3′ and 5′–GTGGTTCTGTGCCCTCCGTGGGTGAAA-3′ and mouse SorCS2 with 5′ – CAGAGGAGCTGCTTGTGACTGTGGTAA – 3′ and 5′ – CACGGGTCTCAGAGGGTGTGTCATT – 3′. Human GAPDH was amplified using the primer pairs 5′-ACCATGGAGAAGGCTGGGGCTCATTT–3′ and 5′–ATGGCATGGACTGTGGTCATGAGTCCTT-3′. Amplified transcripts were separated on a 1.5% agarose gel and quantification of bands performed with ImageJ software (https://imagej.net/ij/). Relevant bands were cut out and purified with RNA NucleoSpin kit (MACHEREY-NAGEL). Sequencing of human SorCS2 was performed with the following primers 5′–ATCAGCTCACCAGGTAGCTGTG–3′ or 5′-CAAGGAAGAGGAGCCTCTCGAGTGATA-3′ and mouse SorCS2 with 5′–CCATAGCCGACTTGTATGTGCTTC–3′ at Eurofins Genomics sequencing facility. Quantification of transcripts containing insertion of nucleotides encoding a serine residue was performed by relating the amplitude of three selected nucleotides from the sequencing profile of transcripts with and without the insertion.

### Cell culturing and stress treatments

The HEK293 and hepatocellular carcinoma (HepG2) cell lines were cultured in Dulbecco's modified Eagle's medium (DMEM) (Lonza) and the neuroblastoma (SH-SY5Y) cell line in DMEM F12 (Lonza), all supplied with 10% fetal calf serum (FCS) (Gibco). To promote UV stress, cells were subjected to radiation from an Osram Puritec hns 15 W, G13 UV lamp at a distance of 50 cm for 15 min and harvested 24 h later. Mild heat stress was induced at 42 °C at 2 h prior to harvest. Hypoosmotic stress was induced by addition of 70% distilled water to the culture medium of the indicated cell lines. Cultures of mouse hippocampal neurons were treated in a similar manner at 6 days in vitro for 24 h with 5 to 30% distilled water added to the culture medium. Transient transfection was done with FuGENE 6 (Promega) using 3.2 μl/ml FuGENE and 0.8 μg/ml cDNA.

### Pull-down experiments

For preparation of HEK293 cell lysate, confluent cells were lysed in TNE lysis buffer (10 mM Tris, pH 8, 150 nM NaCl, 1 mM EDTA) supplemented with 1% NP40 and complete protease inhibitor cocktail (Roche) and cleared at 2000*g* for 5 min. For preparation of human brain homogenate, tissue was homogenized with an ULTRA-TURRAX homogenizer in lysis buffer (20 mM Hepes pH 7.6, 125 mM KAc, 2.5 mM MgAc, 320 mM sucrose, 0.1 mM EDTA) supplemented with complete protease inhibitor cocktail and cleared at 1000*g* for 10 min. The homogenate was then incubated with 1.25% Triton X-100 for 1 h at 4 °C and subsequently cleared by two centrifugation steps, first at 20,000*g* for 15 min and then at 100,000*g* for 1 h.

Hundred micrograms of the cytosolic domain of SorCS2A, SorCS2B, SorCS2C, SorCS2D, sortilin, or DYNLT3 fused to GST was incubated with HEK293 cell lysate or brain homogenate overnight at 4 °C. GST-fusion proteins were then precipitated with glutathione-sepharose beads for 3 h at 4 °C and unspecific binding removed by washing five times for 5 min with lysis buffer. Proteins were eluted by boiling samples in reducing sample buffer (20 mM dithioerythritol, 2.5% SDS) and proteins separated on NuPAGE Bis-Tris 4 to 12% gradient gels from Invitrogen. Antibodies against adaptin γ (610385), μ2 (611350), adaptin δ (611328), and adaptin ε (612018) were all from BD transduction Laboratories. Anti-GST (27457701V) was from GE Healthcare, anti-mouse SorCS2 (AF4237) from R&D Systems, and anti-KLC1 (ab174273) from Abcam. Anti-extracellular SorCS2 F7100 antibody is custom made from DAKO. Images were acquired with the Fujifilm las4000 imaging apparatus using appropriate horseradish peroxidase-conjugated secondary antibodies from DAKO. Further details on the use and performance of antibodies were shared on http://www.pabmabs.com posted by S. Skeldal and S. Glerup.

### Immunofluorescence

HEK293 cells–expressing SorCS2 variants were seeded onto poly-L lysine–coated coverslips 24 h prior to analysis. Surface-exposed SorCS2 was labeled with 1 μg/ml purified antiextracellular SorCS2 F7100 antibody (custom made from DAKO) for 2 h on ice, washed in ice-cold complete medium, and chased at 37 °C in complete medium. Cells were then fixed in 4% paraformaldehyde at indicated time points, permeabilized in 0.1% Triton X-100, and blocked in 10% FCS. Secondary antibody was Alexa-conjugated donkey anti-rabbit 488 (A21206) from Molecular probes, and imaging was performed on a Zeiss LSM780 confocal microscope. For costaining of TGN, cells were incubated with anti-TGN46 (1:100) after permeabilization and blocking and detected with Alexa-conjugated donkey anti-sheep 568 (A21099) from Molecular probes. Zeiss Zen software (https://www.zeiss.com/microscopy/en/products/software/zeiss-zen-lite.html) was used to analyze confocal images and quantify pixel-intensity weighted colocalization coefficients (49).

### Y2H screen

A commercial Y2H screen was performed by Hybrigenics (http://www.hybrigenics.com) to identify APs targeting the ICD of SORCS2D. The ICD of SorCS2D^K1101–S1174^ was cloned into the Hybrigenics bait vector pB27 in fusion with LexA. An adult human brain random-primed cDNA library, transformed into the Y187 yeast strain was used for mating. To certify data from the primary screening, library and bait plasmids were purchased from Hybrigenics for additional verification using control plasmids without inserts. Successfully transformed cells were suspended in sterile ddH2O and spot-plated (5 μl/spot) on plates prepared with 46 g/l yeast minimal agar SD base (Clontech) and 100 μg/ml Pen/Strep (Gibco), and supplemented with either 640 mg/l Leu/Trp dropout supplement (Clontech) for control plates or 640 mg/l Leu/Trp/His dropout supplement (Clontech) for test plates. 3-aminotriazole (Sigma-Aldrich) was added to the plates to suppress bait autoactivation. SORCS2D dialanine mutations were created by overlapping PCR reactions. SorCS2A, SorCS2B, and SorCS2C cytoplasmic PCR fragments were inserted into pB27.

### Pulse-chase experiments

HEK293 cells were seeded into poly-L lysine-coated wells and transiently transfected 24 h later. 24 h post transfection, medium was exchanged with cysteine and methionine free DMEM (Sigma) containing 2% dialyzed FCS and 1% Glutamax for 15 min. Cells were biolabeled for 4 h with ∼300 μCi/ml L-[^35^S]-cysteine and L-[^35^S]-methionine (Pro-mix, Amersham) in the same medium and also supplemented with 10 mg/ml brefeldin A. After a quick wash, cells were chased in complete medium and subsequently lysed at indicated time points in TNE lysis buffer (10 mM Tris, pH 8, 150 nM NaCl, 1 mM EDTA) supplemented with 1% NP40 and complete protease inhibitor cocktail. Samples were immunoprecipitated overnight at 4 °C by use of Gammabind G-Sepharose beads (Amersham) coupled with anti-SorCS2 F7100 antibody. Immunoprecipitates were separated on NuPage Bis-Tris 4 to 12% gradient gels from Invitrogen and processed for fluorography using 2,5-diphenyloxazole.

### Neuronal branching assay

SorCS2^−/−^ postnatal day 0 mouse pups of either sex (9) were euthanized by decapitation, brains removed, and hippocampi dissected into ice-cold Leibowitz’s L-15 medium (Life technologies). Hippocampi were dissociated for 30 min using 20 U/ml pre-activated papain (Bionorica) and washed in DMEM containing 0.01 mg/ml DNase 1 (Sigma) and 10% FCS before being triturated in the same buffer. Following trituration, DMEM was substituted with Neurobasal-A medium supplemented with B-27 supplement, 2 mM GlutaMAX (all from Gibco), 100 μg/ml primocin (Invivogen), 20 μM floxuridine, and 20 μM uridine (both from Sigma). Cells were seeded onto precoated coverslips (poly-D-lysine and laminin [InvivoGen]) at a density of 100,000/coverslip and incubated at 37 °C for 24 h. Medium was then substituted with Neurobasal-A medium without antibiotics and transfected using lipofectamine (InvivoGen). For each cotransfection, 125 ng of plasmid encoding SorCS2 and 125 ng plasmid encoding GFP were added to 100 μl Opti-MEM reduced serum medium (Gibco) and 0.25 μl plus reagent added to the diluted DNA and incubated at room temperature for 5 min. Hereafter, 1.25 μl lipofectamine LTX was added, and the solution was incubated at room temperature for 30 min. The DNA–lipid complex solution was then added to neurons and medium substituted with Neurobasal-Amedium with or without 1 nM BDNF (Millipore) after 6 h at 37 °C.

After 72 h, neurons were fixed with 4% paraformaldehyde, and images were obtained using confocal microscopy. Branching pattern of GFP-positive neurons was analyzed using Zen 2011 image processing (https://www.zeiss.com/microscopy/en/products/software/zeiss-zen-lite.html). At least ten neurons were analyzed on three or four coverslips for each condition. The use of animals in this study has been approved by the Danish Animal Experiments Expectorate (License file number 2016-15-0202-00050).

### RNA sequencing analysis

Data files were downloaded from Sequence Read Archive using SRAToolkit. For transcript quantification, Kallisto software (https://pachterlab.github.io/kallisto/about.html) was used to normalize to the human genome hg38. For differential expression analysis of *SORCS2* in autism and schizophrenia patients *versus* controls the R package DESeq2 (http://www.bioconductor.org/packages/release/bioc/html/DESeq2.html) was used to normalize Kallisto output.

### Data analysis and statistics

Statistical analyses were performed using unpaired two-tailed Student’s *t* test unless otherwise stated. Variances in *SorCS2* mRNA levels in patients suffering from autism or schizophrenia and control groups were compared using an F-test. BDNF treatment responses in different groups of hippocampal neurons were compared by two-way ANOVA. If the analysis of variance revealed significant differences among groups, a post hoc test (unpaired two-tailed Student’s *t* test) was carried out to elucidate the pattern of differences.

Values are presented as mean ± SD and the significance level was set at *p* < 0.05.

## Data availability

The authors declare that all data supporting the findings of this study are available within the paper. The nucleotide sequence encoding human SorCS2A, SorCS2B, SorCS2C, and SorCS2D have been deposited at the GenBank sequence database (accession number OQ616758, OQ616759, OQ616760, and OQ616761, respectively).

## Conflict of interest

The authors declare that they have no conflicts of interest with the contents of this article.

## References

[1] Kelemen O., Convertini P., Zhang Z., Wen Y., Shen M., Falaleeva M. (2013). Function of alternative splicing. Gene.

[2] Yang X., Coulombe-Huntington J., Kang S., Sheynkman G.M., Hao T., Richardson A. (2016). Widespread expansion of protein interaction capabilities by alternative splicing. Cell.

[3] Caceres J.F., Kornblihtt A.R. (2002). Alternative splicing: multiple control mechanisms and involvement in human disease. Trends Genet..

[4] Rezgaoui M., Hermey G., Riedel I.B., Hampe W., Schaller H.C., Hermans-Borgmeyer I. (2001). Identification of SorCS2, a novel member of the VPS10 domain containing receptor family, prominently expressed in the developing mouse brain. Mech. Dev..

[5] Nagase T., Kikuno R., Ishikawa K., Hirosawa M., Ohara O. (2000). Prediction of the coding sequences of unidentified human genes. XVII. The complete sequences of 100 new cDNA clones from brain which code for large proteins *in vitro*. DNA Res..

[6] Glerup S., Nykjaer A., Vaegter C.B. (2014). Sortilins in neurotrophic factor signaling. Handb. Exp. Pharmacol..

[7] Hermey G., Plath N., Hubner C.A., Kuhl D., Schaller H.C., Hermans-Borgmeyer I. (2004). The three sorCS genes are differentially expressed and regulated by synaptic activity. J. Neurochem..

[8] Boggild S., Molgaard S., Glerup S., Nyengaard J.R. (2016). Spatiotemporal patterns of sortilin and SorCS2 localization during organ development. BMC Cell Biol..

[9] Glerup S., Olsen D., Vaegter C.B., Gustafsen C., Sjoegaard S.S., Hermey G. (2014). SorCS2 regulates dopaminergic wiring and is processed into an apoptotic two-chain receptor in peripheral glia. Neuron.

[10] Deinhardt K., Kim T., Spellman D.S., Mains R.E., Eipper B.A., Neubert T.A. (2011). Neuronal growth cone retraction relies on proneurotrophin receptor signaling through Rac. Sci. Signal..

[11] Glerup S., Bolcho U., Molgaard S., Boggild S., Vaegter C.B., Smith A.H. (2016). SorCS2 is required for BDNF-dependent plasticity in the hippocampus. Mol. Psychiatry.

[12] Ma Q., Yang J., Milner T.A., Vonsattel J.G., Palko M.E., Tessarollo L. (2017). SorCS2-mediated NR2A trafficking regulates motor deficits in Huntington's disease. JCI Insight.

[13] Baum A.E., Akula N., Cabanero M., Cardona I., Corona W., Klemens B. (2008). A genome-wide association study implicates diacylglycerol kinase eta (DGKH) and several other genes in the etiology of bipolar disorder. Mol. Psychiatry.

[14] Christoforou A., McGhee K.A., Morris S.W., Thomson P.A., Anderson S., McLean A. (2011). Convergence of linkage, association and GWAS findings for a candidate region for bipolar disorder and schizophrenia on chromosome 4p. Mol. Psychiatry.

[15] Ollila H.M., Soronen P., Silander K., Palo O.M., Kieseppa T., Kaunisto M.A. (2009). Findings from bipolar disorder genome-wide association studies replicate in a Finnish bipolar family-cohort. Mol. Psychiatry.

[16] Alemany S., Ribases M., Vilor-Tejedor N., Bustamante M., Sanchez-Mora C., Bosch R. (2015). New suggestive genetic loci and biological pathways for attention function in adult attention-deficit/hyperactivity disorder. Am. J. Med. Genet. B Neuropsychiatr. Genet..

[17] Reitz C., Tosto G., Vardarajan B., Rogaeva E., Ghani M., Rogers R.S. (2013). Independent and epistatic effects of variants in VPS10-d receptors on Alzheimer disease risk and processing of the amyloid precursor protein (APP). Transl. Psychiatry.

[18] Smith A.H., Ovesen P.L., Skeldal S., Yeo S., Jensen K.P., Olsen D. (2018). Risk locus identification ties alcohol withdrawal symptoms to SORCS2 alcohol. Clin. Exp. Res..

[19] Olsen D., Kaas M., Lundhede J., Molgaard S., Nykjaer A., Kjolby M. (2019). Reduced alcohol seeking and withdrawal symptoms in mice lacking the BDNF receptor SorCS2. Front. Pharmacol..

[20] Anastasia A., Deinhardt K., Chao M.V., Will N.E., Irmady K., Lee F.S. (2013). Val66Met polymorphism of BDNF alters prodomain structure to induce neuronal growth cone retraction. Nat. Commun..

[21] Bonifacino J.S., Traub L.M. (2003). Signals for sorting of transmembrane proteins to endosomes and lysosomes. Annu. Rev. Biochem..

[22] Braulke T., Bonifacino J.S. (2009). Sorting of lysosomal proteins. Biochim. Biophys. Acta.

[23] Baltes J., Larsen J.V., Radhakrishnan K., Geumann C., Kratzke M., Petersen C.M. (2014). sigma1B adaptin regulates adipogenesis by mediating the sorting of sortilin in adipose tissue. J. Cell Sci..

[24] Nielsen M.S., Gustafsen C., Madsen P., Nyengaard J.R., Hermey G., Bakke O. (2007). Sorting by the cytoplasmic domain of the amyloid precursor protein binding receptor SorLA. Mol. Cell. Biol..

[25] Traunmuller L., Gomez A.M., Nguyen T.M., Scheiffele P. (2016). Control of neuronal synapse specification by a highly dedicated alternative splicing program. Science.

[26] Wang P., Yan B., Guo J.T., Hicks C., Xu Y. (2005). Structural genomics analysis of alternative splicing and application to isoform structure modeling. Proc. Natl. Acad. Sci. U. S. A..

[27] Romero P.R., Zaidi S., Fang Y.Y., Uversky V.N., Radivojac P., Oldfield C.J. (2006). Alternative splicing in concert with protein intrinsic disorder enables increased functional diversity in multicellular organisms. Proc. Natl. Acad. Sci. U. S. A..

[28] Bonifacino J.S. (2014). Adaptor proteins involved in polarized sorting the. J. Cell Biol..

[29] Farias G.G., Cuitino L., Guo X., Ren X., Jarnik M., Mattera R. (2012). Signal-mediated, AP-1/clathrin-dependent sorting of transmembrane receptors to the somatodendritic domain of hippocampal neurons. Neuron.

[30] Blom N., Gammeltoft S., Brunak S. (1999). Sequence and structure-based prediction of eukaryotic protein phosphorylation sites. J. Mol. Biol..

[31] Blom N., Sicheritz-Ponten T., Gupta R., Gammeltoft S., Brunak S. (2004). Prediction of post-translational glycosylation and phosphorylation of proteins from the amino acid sequence. Proteomics.

[32] Deinhardt K., Chao M.V. (2014). Trk receptors. Handb. Exp. Pharmacol..

[33] Minichiello L. (2009). TrkB signalling pathways in LTP and learning. Nat. Rev. Neurosci..

[34] Biamonti G., Caceres J.F. (2009). Cellular stress and RNA splicing. Trends Biochem. Sci..

[35] Bringmann A., Uckermann O., Pannicke T., Iandiev I., Reichenbach A., Wiedemann P. (2005). Neuronal versus glial cell swelling in the ischaemic retina. Acta Ophthalmol. Scand..

[36] Berk B.A., Vogler S., Pannicke T., Kuhrt H., Garcia T.B., Wiedemann P. (2015). Brain-derived neurotrophic factor inhibits osmotic swelling of rat retinal glial (Muller) and bipolar cells by activation of basic fibroblast growth factor signaling. Neuroscience.

[37] Vogler S., Hollborn M., Berk B.A., Pannicke T., Seeger J., Wiedemann P. (2016). Ischemic regulation of brain-derived neurotrophic factor-mediated cell volume and TrkB expression in glial (Muller) and bipolar cells of the rat retina. Graefes Arch. Clin. Exp. Ophthalmol..

[38] Guedes-Dias P., Holzbaur E.L.F. (2019). Axonal transport: driving synaptic function. Science.

[39] Hirokawa N., Niwa S., Tanaka Y. (2010). Molecular motors in neurons: transport mechanisms and roles in brain function, development, and disease. Neuron.

[40] Reck-Peterson S.L., Redwine W.B., Vale R.D., Carter A.P. (2018). The cytoplasmic dynein transport machinery and its many cargoes. Nat. Rev. Mol. Cell Biol..

[41] Fan R., Lai K.O. (2022). Understanding how kinesin motor proteins regulate postsynaptic function in neuron. FEBS J..

[42] Moya-Alvarado G., Guerra M.V., Tiburcio R., Bravo E., Bronfman F.C. (2022). The Rab11-regulated endocytic pathway and BDNF/TrkB signaling: roles in plasticity changes and neurodegenerative diseases. Neurobiol. Dis..

[43] Arimura N., Kimura T., Nakamuta S., Taya S., Funahashi Y., Hattori A. (2009). Anterograde transport of TrkB in axons is mediated by direct interaction with Slp1 and Rab27. Dev. Cell.

[44] Alsabban A.H., Morikawa M., Tanaka Y., Takei Y., Hirokawa N. (2020). Kinesin Kif3b mutation reduces NMDAR subunit NR2A trafficking and causes schizophrenia-like phenotypes in mice. EMBO J..

[45] Muresan Z., Muresan V. (2005). Coordinated transport of phosphorylated amyloid-beta precursor protein and c-Jun NH2-terminal kinase-interacting protein-1. J. Cell Biol..

[46] Kamal A., Stokin G.B., Yang Z., Xia C.H., Goldstein L.S. (2000). Axonal transport of amyloid precursor protein is mediated by direct binding to the kinesin light chain subunit of kinesin-I. Neuron.

[47] Kaerlev L., Kolstad H.A., Hansen A.M., Thomsen J.F., Kaergaard A., Rugulies R. (2011). Are risk estimates biased in follow-up studies of psychosocial factors with low base-line participation?. BMC Public Health.

[48] Kolstad H.A., Hansen A.M., Kaergaard A., Thomsen J.F., Kaerlev L., Mikkelsen S. (2011). Job strain and the risk of depression: is reporting biased?. Am. J. Epidemiol..

[49] Manders E.M.M., Verbeek F.J., Aten J.A. (1993). Measurement of co-localization of objects in dual-colour confocal images. J. Microsc..

